# Intravenous Thrombolysis Prior to Endovascular Treatment in Basilar Artery Occlusions: A Patient Pooled Analysis of Four Randomized Controlled Trials

**DOI:** 10.1007/s00270-025-04251-z

**Published:** 2025-10-27

**Authors:** Robrecht R. M. M. Knapen, Mohamed F. Doheim, Heinrich. J. Audebert, Francisco Mont’Alverne, Volker Pütz, Patrik Michel, Xinfeng Liu, Qiliang Dai, Wei Hu, Chunrong Tao, Rui Liu, Pengfei Xu, Chuanhui Li, Xunming Ji, Tudor G. Jovin, Christiaan van der Leij, Robert J. van Oostenbrugge, Wim H. van Zwam, Raul G. Nogueira, Wouter J. Schonewille

**Affiliations:** 1 Department of Radiology and Nuclear Medicine, Maastricht University Medical Center+, P. Debyelaan 25, 6229HX Maastricht, The Netherlands; 2 School for Cardiovascular Diseases Maastricht (CARIM), Maastricht University, Maastricht, The Netherlands; 3 Department of Neurology, UPMC Stroke Institute, University of Pittsburgh, Pittsburgh, PA USA; 4 Department of Neurology and Center for Stroke Research, Charité Universitätsmedizin Berlin, Berlin, Germany; 5 Interventional Neuroradiology Service, Hospital Geral de Fortaleza, Fortaleza, Brazil; 6 Department of Neurology, Dresden Neurovascular Center, University Clinics Carl Gustav Carus, Technische Universität Dresden, Dresden, Germany; 7 Stroke Center, Neurology Service, Lausanne University Hospital and University of Lausanne, Lausanne, Switzerland; 8 Department of Neurology, Division of Life Science and Medicine, The First Affiliated Hospital of USTC, University of Science and Technology of China, Hefei, Anhui China; 9 Department of Neurology, Nanjing Jinling Hospital, Nanjing University, Nanjing, China; 10 Stroke Center, Department of Neurology, Xuanwu Hospital of Capital Medical University, Beijing, China; 11 Department of Neurosurgery, Xuanwu Hospital of Capital Medical University, Beijing, China; 12 Department of Neurology, Cooper University Healthcare and Cooper Medical School of Rowan University, Camden, USA; 13 Research Institute for Oncology and Reproduction (GROW), Maastricht University, Maastricht, The Netherlands; 14 Department of Neurology, Maastricht University Medical Center+, Maastricht, The Netherlands; 15 Department of Neurology, St. Antonius Hospital, Nieuwegein, The Netherlands

**Keywords:** Stroke, Basilar artery occlusion, BAO, Intravenous thrombolysis, IVT, BASICS, BEST, BAOCHE, ATTENTION

## Abstract

**Purpose:**

This study assessed intravenous thrombolysis (IVT) prior to endovascular treatment (EVT) versus EVT alone in patients with basilar artery occlusions (BAO).

**Methods:**

This patient-level pooled analysis included data from four randomized controlled trials within the VERITAS collaboration (BEST, BASICS, ATTENTION, and BAOCHE). Patients were stratified into IVT plus EVT and EVT alone. Primary outcome was favorable functional outcome, defined as modified Rankin Scale (mRS) score of 0–3 at 3 months. Secondary outcomes included good functional outcome (mRS 0–2), mortality, and symptomatic intracranial hemorrhage (sICH) rates. Regression analyses were adjusted for covariates identified from baseline differences and univariable analyses. Inverse probability of treatment weighting (IPTW) and propensity score matching (PSM) to balance baseline differences and subgroup analyses were also conducted.

**Results:**

Out of total 988 included patients, 556 patients were allocated for EVT and analyzed. No significant differences were observed between patients treated with or without IVT prior to EVT in terms of mRS 0–3 at 3 months (47 vs 44%, adjusted odds ratio [aOR]:0.88, 95%CI 0.57–1.36), mRS 0–2 (39 vs 32%, aOR:1.22, 95%CI 0.78–1.91), mortality (33 vs 38%, aOR:0.93, 95%CI 0.59–1.44), and sICH rates (6.3 vs 4.9%, aOR:1.87, 95%CI 0.77–4.57). IPTW and PSM analyses yielded consistent results. Subgroup analyses did not reveal any differential treatment effect including time from symptom onset to imaging.

**Conclusions:**

Findings from this patient-level pooled analysis of four randomized controlled trials suggest that bridging IVT over EVT alone was safe but not associated with significant improved outcomes.

**Level of evidence 2b:**

Level 2b, cohort study of a patient-level meta-analysis of 4 RCT’s.

**Graphical Abstract:**

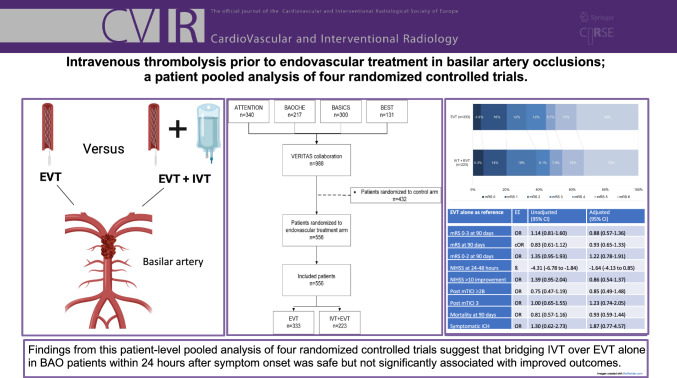

**Supplementary Information:**

The online version contains supplementary material available at 10.1007/s00270-025-04251-z.

## Introduction

The use of intravenous thrombolysis (IVT) prior to endovascular treatment (EVT) is recommended by the European Stroke Organisation (ESO) guidelines in ischemic stroke patients with large vessel occlusion within 4.5 h from symptom onset [1]. The IRIS collaboration, encompassing six randomized controlled trials (RCTs), could not establish non-inferiority of EVT alone compared to bridging therapy with IVT in patients presenting directly at endovascular treatment centers [2]. Knowledge on the use of IVT prior to EVT in patients with posterior circulation stroke due to large vessel occlusion is limited. Despite this, the latest ESO and European Society for Minimally Invasive Neurological Therapy (ESMINT) guidelines recommend IVT before EVT in posterior circulation stroke [3].

A meta-analysis suggested benefit from IVT in posterior circulation stroke especially if initiated within 4.5 h from symptom onset [4]. However, the question arose whether this benefit is still present in patients treated with EVT. The BEST, BASICS, ATTENTION, and BAOCHE trials were RCTs on the effectiveness of EVT in patients with basilar artery occlusion (BAO) [5–8]. Pooled data of the four trials in the VERITAS study highlighted the strong benefit of EVT in patients with BAO and moderate to severe symptoms [9].

Bridging IVT before EVT was investigated by multiple cohort studies, including a post hoc analysis of the ATTENTION trial and a MR CLEAN Registry study, showing comparable results between IVT and no IVT prior to EVT for posterior circulation strokes [10–12]. Nevertheless, patients, who recovered from IVT before EVT within the MR CLEAN Registry, were not taken into account, potentially underestimating the value of IVT in the posterior circulation. A meta-analysis concluded better clinical outcomes with equal incidence of symptomatic intracranial hemorrhage (sICH) in patients bridged with IVT; however, most included studies were cohort studies [13].

The VERITAS collaboration did not describe a significant interaction effect of IVT with EVT on the functional outcome for BAO patients [9]. The aim of this study was to assess the influence of IVT prior to EVT on the outcome in BAO patients.

## Methods

### Design and Participants

The VERITAS collaboration performed a patient-level meta-analysis of four RCTs that investigated the efficacy and safety of EVT in BAO [9]. The search strategy, selection process, data collection process, and statistical analysis plan were published earlier [9]. The inclusion criteria of the BAOCHE, BEST, BASICS, as well as the ATTENTION Trial were published earlier [5–8]. All trials randomized BAO patients to either the intervention arm (receiving EVT in addition to best medical care) and the control arm (best medical care), with a range from symptom onset or estimated time of BAO until randomization of 0–24 h. All patients were treated according to the study protocol and national guidelines, including the administration of IVT when indicated. Among the four trials, r-tPA (recombinant tissue-type plasminogen activator; Alteplase) and uPa (urokinase-type plasminogen activator; Urokinase) were both used as IV thrombolytics.

Data were collected from each trial and were part of the VERITAS database. After collation of the data, data were compared to the published data of each trial. Only minor discrepancies within baseline characteristics were objectivated, and these were resolved in collaboration with trial investigators. In this study, all patients from the intention-to-treat population were stratified into two groups: IVT plus EVT and EVT alone. Patients from the control arm were excluded. This study was conducted using the STROBE guidelines [14].

### Outcome Measures

The primary outcome was favorable functional outcome defined as a score of 0–3 on the modified Rankin Scale (mRS) 90-day follow-up. The mRS ranges from 0 (no disability) to 6 (death). Secondary outcomes included good functional outcome (mRS 0–2), shift analysis of mRS score at 90 days, the National Institute of Health Stroke Scale (NIHSS) score at 24 h, early neurological recovery (defined as at least a ten-point decrease in NIHSS score at 24 h), and reperfusion grade (modified Thrombolysis In Cerebral Infarction [mTICI] score).

The safety outcomes were 90-day mortality and symptomatic intracranial hemorrhage (sICH) at 24 h. sICH was primarily assessed based on the definition from the Safe Implementation of Thrombolysis in Stroke–Monitoring Study (SITS-MOST), which included parenchymal hemorrhage type 2 on follow-up imaging and a neurological deterioration of at least 4 points on the NIHSS. When the SITS-MOST definition was unavailable, we used the definition from the second European Cooperative Acute Stroke Study (ECASS II), which considered any type of intracranial hemorrhage on post-treatment imaging accompanied by an increase of at least 4 points on the NIHSS, with the hemorrhage deemed the primary cause of neurological deterioration [15, 16].

### Statistical Analysis

Baseline characteristics were compared between patients treated with IVT and without IVT prior EVT using descriptive statistics. The primary outcome was analyzed using multivariable logistic regression analysis. Secondary outcomes were analyzed using ordinal, binary, or linear regression analyses as appropriate. All analyses were performed based on the intention-to-treat-principle and with EVT alone as reference. For all analyses, the (common) odds ratio ([c]OR) or the beta-estimates with their 95%CI confidence interval (CI) was presented. Regression models were adjusted for potential confounders; these variables were chosen based on baseline differences, literature, and univariable analyses. Analyses were adjusted for: age, baseline pc-ASPECTS on CT, CT angiography (CTA), or magnetic resonance imaging (MRI), baseline NIHSS score, atrial fibrillation, hypertension, TOAST classification, occlusion location, and onset to imaging time. A random intercept for trial effect was considered when statistically significant.

Four prespecified secondary analyses were performed. The first analysis was an inverse probability of treatment weighting (IPTW) with propensity score method analysis to balance both treatment groups based on the baseline differences. The regression analyses after IPTW were adjusted based on univariable analyses (age and baseline NIHSS). Second, a sensitivity analysis was performed after removing patients with symptom onset to imaging > 270 min to investigate the influence of bridging IVT on clinical outcome based on the 4.5 h recommendation of IVT in the anterior circulation by the ESO guidelines [1]. Based on the VERITAS main paper, we used the symptom onset to imaging time to determine the duration of BAO [9]. Third, a propensity score matching (PSM) analysis was performed to investigate the effect of IVT on outcome after EVT in a matched patient population based on baseline characteristics. Fourth, treatment effect heterogeneity was tested on the primary outcome for all clinically relevant confounders and presented using forest plots along with the p value of the interaction term (IVT*confounder). All statistical analyses were performed using R (Version 4.3.1). The significance level was set at 5%.

### Missing Values

There was no missing data in the primary outcome. Missing baseline data were imputed with multiple imputations with chained equations (MICE) using the *mice* package (version 3.16.0), before adding in the regression models. The number of imputations was set at 50.

## Results

### Baseline Characteristics

**Fig. 1:**
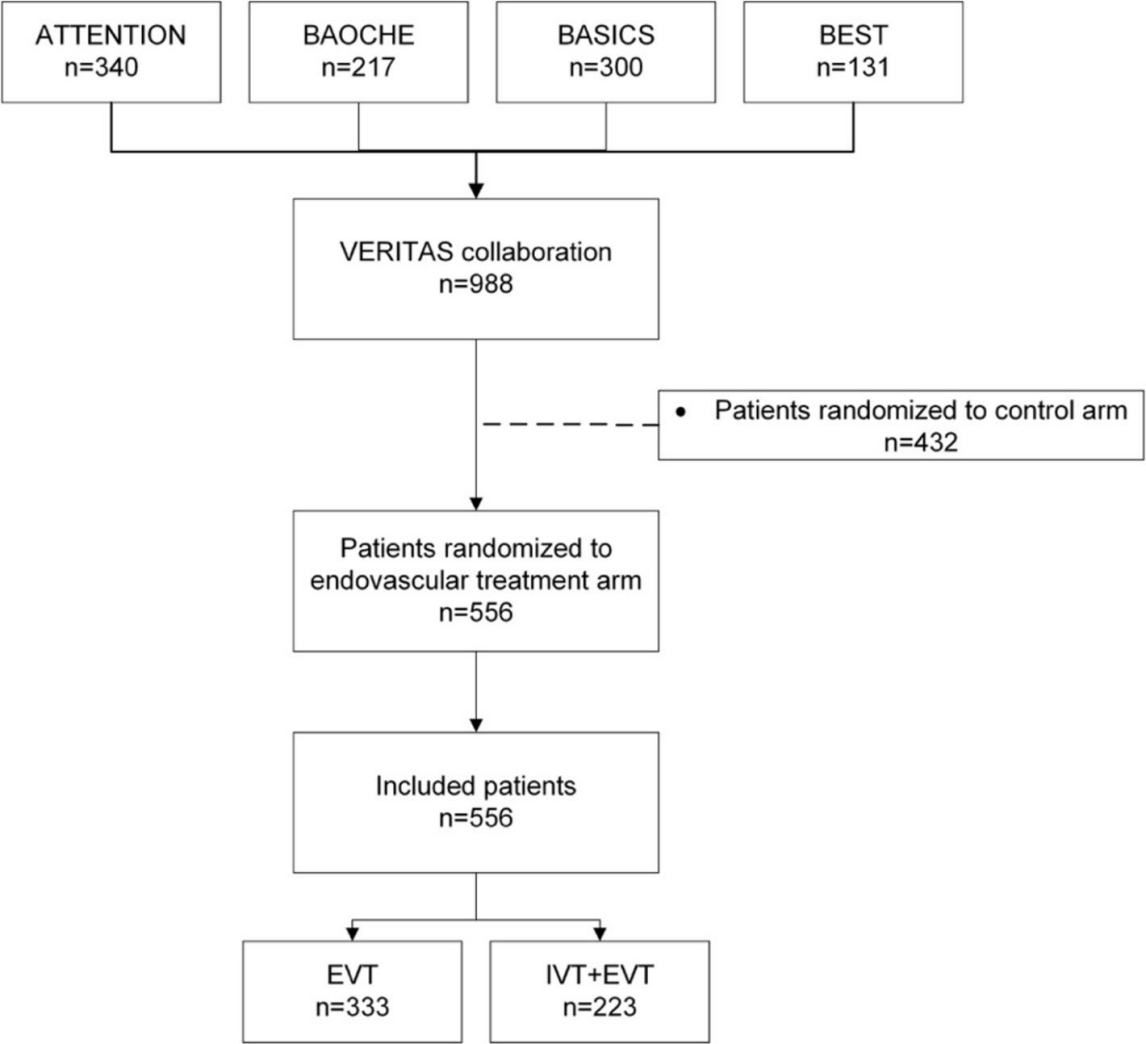
Flowchart of included patients. EVT, endovascular treatment; IVT intravenous thrombolysis

A total of 988 patients were pooled from the four trials included in the VERITAS collaboration. After excluding all patients randomized to control arm, all 556 patients from the EVT group were included in this analysis of whom 333 patients received EVT without IVT and 223 patients received EVT and IVT (Fig. 1). Baseline characteristics are overviewed in Table 1.

**Table 1:** Baseline characteristics of included patients

	EVT (*n* = 333)		EVT + IVT (*n* = 223)		Missing (*n* =)	*P*-value
Age—Mean (SD)	64.6	(11.8)	66.4	(12.3)	0	0.09
Male—No. (%)	235	(71)	142	(64)	0	0.11
*Patients history—No. (%)*						
Previous stroke	71	(21)	39	(18)	0	0.32
Atrial fibrillation	52	(16)	69	(31)	0	**< 0.001**
Hypertension	252	(76)	139	(62)	0	**< 0.01**
Diabetes Mellitus	79	(24)	44	(20)	1	0.33
CAD	51	(15)	31	(14)	0	0.74
*Clinical*						
NIHSS baseline—Median [IQR]*	23	[15–35]	22	[12–35]	0	0.19
Pre-mRS—No. (%)					1	0.45
0	278	(84)	191	(86)		
1	41	(12)	20	(9.0)		
2	14	(4.2)	11	(5.0)		
TOAST—No. (%)					8	**< 0.001**
LAA	185	(56)	88	(41)		
CE	52	(16)	71	(33)		
SVD + other/un-determined	96	(29)	56	(26)		
*Imaging*						
Occlusion location—No. (%)					79	**< 0.01**
None	1	(0.4)	0	(0.0)		
Intracranial VA	27	(9.7)	5	(2.5)		
BA proximal	99	(36)	57	(29)		
BA middle	83	(30)	68	(34)		
BA distal	69	(25)	68	(34)		
pc-ASPECTS—median [IQR]	9	[7–10]	10	[8–10]	12	** < 0.001**
*Procedure*						
Duration onset symptoms to imaging in minutes—median [IQR]	320	[186–493]	120	[67.3–199]	31	**< 0.001**

Bold value indicates *p* < 0.001

*EVT* endovascular treatment; *IVT* intravenous thrombolysis; *CAD* coronary artery disease; *NIHSS* National Institutes of Health Stroke Scale; *mRS* modified Rankin Scale; *TOAST* Trial of Org 10,172 in Acute Stroke Treatment; *LAA* large-artery atherosclerosis; *CE* cardioembolic; *SVD* small vessel disease; *VA* vertebral artery; *BA* basilar artery; *pc-ASPECTS* posterior circulation Acute Stroke Prognosis Early Computed Tomography score; *DSA* digital subtraction angiography

**Fig. 2:**
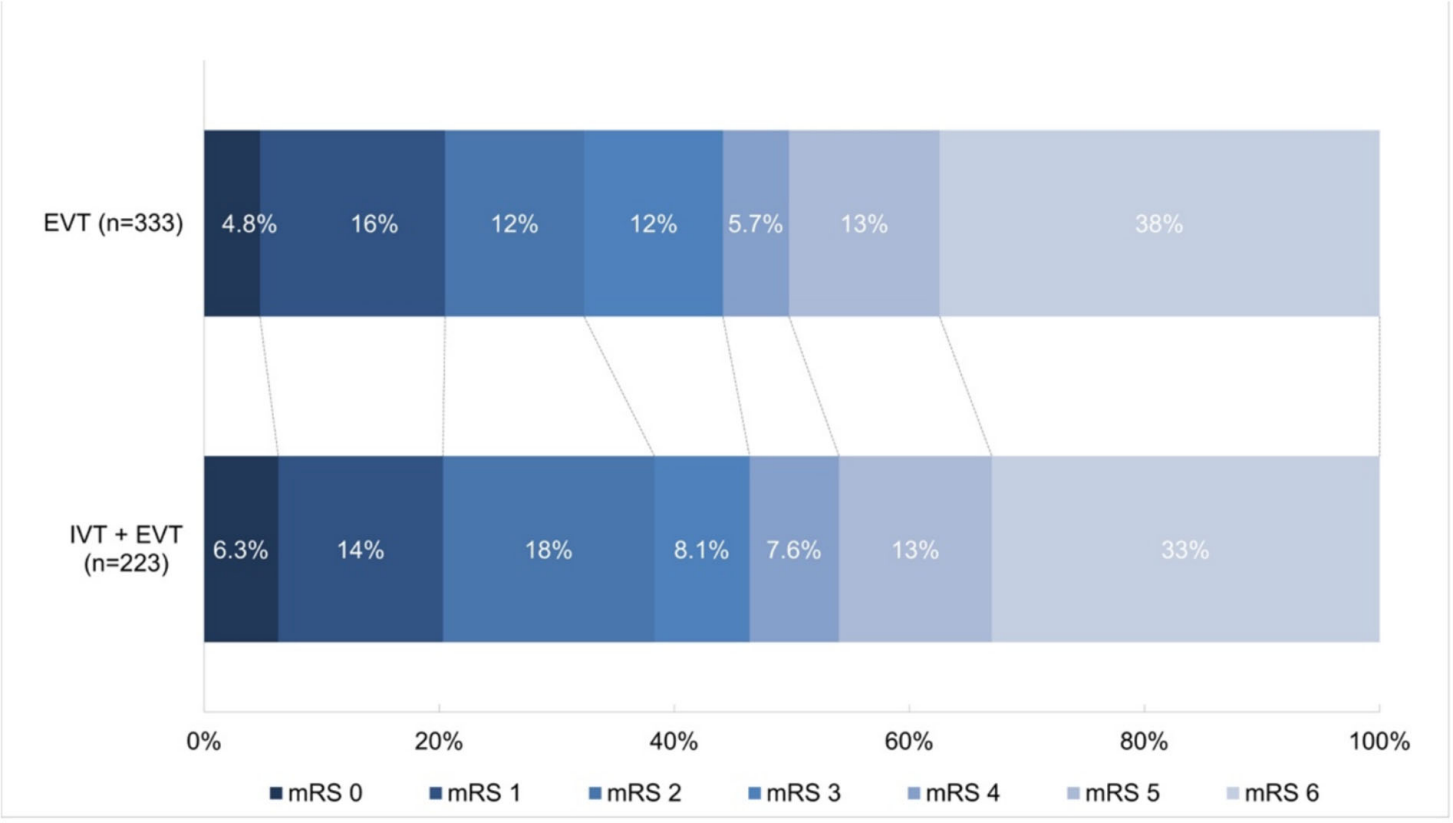
Distribution of mRS score at 90-day follow-up between both groups. EVT, endovascular treatment; IVT, intravenous thrombolysis; mRS, modified Rankin Scale

At 90-day follow-up, the proportion of patients with a favorable functional outcome did not differ between those treated with or without IVT prior to EVT (47 vs. 44%; aOR:0.88, 95%CI 0.57–1.36) (Tables 2, and 3). Figure 2 illustrates the distribution of the mRS score at 90 days, showing no differences between both groups (acOR:0.93, 95%CI 0.65–1.33) (Table 3). No statistically significant differences were objectivated among the other secondary outcomes between the groups (Table 3).

**Table 2:** Outcomes between patients treated with and without IVT

	EVT (*n* = 333)		EVT + IVT (*n* = 223)		Missing (*n* =)
*Primary outcome*					
mRS 0–3 at 90 days—No. (%)	146	(44)	105	(47)	0
*Secondary outcomes*					
mRS at 90 days—No. (%)					0
0	16	(4.8)	14	(6.3)	
1	52	(16)	32	(14)	
2	39	(12)	41	(18)	
3	39	(12)	18	(8.1)	
4	19	(5.7)	17	(7.6)	
5	43	(13)	28	(13)	
6	125	(38)	73	(33)	
mRS 0–2 at 90 days—No. (%)	107	(32)	87	(39)	0
NIHSS at 24 h—Median [IQR]	22	[9.0–36]	13	[4.0–35]	27
Post mTICI ≥ 2B—No. (%)	182	(73)	82	(67)	183
Post mTICI 3—No. (%)	136	(54)	67	(55)	183
*Safety outcomes*					
Mortality at 90 days—No. (%)	125	(38)	73	(33)	0
sICH—No. (%)	16	(4.9)	14	(6.3)	8

*EVT* endovascular treatment; *IVT* intravenous thrombolysis; *mRS* modified Rankin Scale; *NIHSS* National Institutes of Health Stroke Scale; *mTICI* modified Thrombolysis In Cerebral Infarction; *sICH* symptomatic intracranial hemorrhage

**Table 3:** Outcomes of regression analyses on clinical, technical, and safety outcomes

EVT alone as reference	EE	Unadjusted (95% CI)	Adjusted (95% CI)
*Primary outcome*			
mRS 0–3 at 90 days	OR	1.14 (0.81–1.60)	0.88 (0.57–1.36)
*Secondary outcomes*			
mRS at 90 days*	cOR	0.83 (0.61–1.12)	0.93 (0.65–1.33)
mRS 0–2 at 90 days	OR	1.35 (0.95–1.93)	1.22 (0.78–1.91)
NIHSS at 24–48 h	ß	− **4.31 (–6.78 to** − **1.84)**	− 1.64 (− 4.13 to 0.85)
NIHSS > 10 improvement	OR	1.39 (0.95–2.04)	0.86 (0.54–1.37)
Post mTICI ≥ 2B	OR	0.75 (0.47–1.19)	0.85 (0.49–1.48)
Post mTICI 3	OR	1.00 (0.65–1.55)	1.23 (0.74–2.05)
*Safety outcomes*			
Mortality at 90 days	OR	0.81 (0.57–1.16)	0.93 (0.59–1.44)
Symptomatic ICH	OR	1.30 (0.62–2.73)	1.87 (0.77–4.57)

Bold value indicates *p* < 0.001

*Shift toward a higher (worse) functional outcome on the full scale

*EE* effect estimate; *OR* odds ratio, *cOR* common odds ratio; *mRS* modified Rankin Scale; *eTICI* extended Thrombolysis In Cerebral Infarction; *ICH* intracranial hemorrhage; *NIHSS* National Institutes of Health Stroke Scale

### IPTW, Sensitivity Analysis, and PSM

IPTW analysis weighted to account for age and baseline NIHSS demonstrated similar trends, with no significant differences in favorable functional outcome, secondary outcomes, or safety outcomes between both groups (Table S1). When selecting only patients who presented within 4.5 h from symptom onset to imaging, no significant differences were observed in primary, secondary, or safety outcomes between both groups (Table S2). No significant differences in primary and secondary outcomes were observed after the PSM analyses (Table S3).

### Subgroup Analysis

Subgroup analyses were conducted to explore the potential influence of confounders on treatment effects. Despite significant interactions between hypertension, baseline NIHSS score, and treatment effect, subgroup analyses revealed no differences in treatment outcomes across the groups. These factors appeared to influence the treatment effect but did not result in varying treatment responses within the subgroups (Fig. S1).

## Discussion

In this pooled analysis of patient-level data from the VERITAS collaboration, we demonstrated no significant differences in clinical outcomes in BAO patients treated with IVT prior to EVT compared to EVT alone. This observation remained consistent in patients with imaging within 4.5 h after symptom onset or estimated time of BAO.

Cohort studies have shown comparable results between patients treated with or without IVT prior to EVT [10–12]. However, a prospective registry study and a meta-analysis concluded higher rates of favorable functional outcome and lower mortality risk, without increased sICH rates in the bridging group [13, 18]. Despite these findings, our pooled analysis demonstrated similar favorable functional outcomes, mortality, and sICH rates, suggesting that IVT may have limited added value but also limited added risk when followed by EVT in BAO patients.

The limited benefit of IVT observed in this analysis may be explained by specific characteristics reported in the BASICS, ATTENTION, BAOCHE, and BEST trials. While 79% of patients in the BASICS in the EVT arm received IVT, this rate was lower in ATTENTION (31%), BAOCHE (14%), and BEST (27%). Importantly, the timing of IVT administration and EVT differed across these trials, which could also influence the observed treatment effects [5–8]. However, favorable functional outcomes in the EVT arm were similar among the four trials, ranging between 42 and 46%. This implies that IVT may not enhance outcomes beyond those achieved with EVT alone in BAO patients. In the control arms of these four trials, differences emerged. In the BASICS trial, a higher proportion of patients in the control group (38%) achieved a mRS 0–3 compared to the other trials (ATTENTION 23%, BAOCHE 24%, and BEST 32%) [5–8]. This discrepancy may be attributed to the higher use of IVT in the control arm of BASICS compared to the others, suggesting that IVT alone may obviate the need for EVT. This effect has been proposed in a cohort study as well [10].

The majority of patients in this study who were treated with IVT prior to EVT were enrolled in the BASICS trial; this may have introduced selection bias. This is primarily due to the time window in BASICS (estimated time of BAO to EVT between 0 and 6 h) [6]. One study reported lower mortality rates in patients treated with bridging therapy when treated within 6 h after symptom onset [18]. Given that time to recanalization is an independent predictor of good outcome in BAO patients, the shorter time from symptom onset to imaging observed in the bridging group in this study may have positively influenced the results [19]. However, after adjusting for time between symptom onset and imaging in IPTW analyses, no significant interaction between the primary outcome and time between symptom onset and imaging was observed. Moreover, when analyzing only patients who presented within 4.5 h of symptom onset to imaging, no significant differences were found in primary, secondary, or safety outcomes between the two groups. Further studies might be required to explore these findings more deeply.

Studies have shown that patients with posterior circulation stroke treated solely with IVT may experience lower sICH rates compared to those with anterior circulation occlusions [20, 21]. Additionally, prior research indicated that administering IVT before EVT in patients with anterior circulation occlusion carries a potential higher sICH risk compared to those not receiving IVT [2]. However, this study and other cohort studies did not find a statistically significant difference in sICH rates suggesting potential better safety of IVT prior to EVT in the posterior circulation [12, 22, 23]. This may be due to the more developed collateral pathways, particularly in the brainstem [24]. Furthermore, a study evaluating the predictive value of the pc-ASPECTS on CT angiography source images for final infarct extent and hemorrhagic transformation (HT) in patients with basilar artery occlusion showed that lower pc-ASPECTS scores correlated with greater final infarct extent while HT rates were higher in patients with lower scores, although thrombolysis use did not significantly impact HT risk [25].

A notable interaction was observed in the subgroup analyses with EVT alone potentially benefiting BAO patients who had arterial hypertension or a higher age. However, the interaction effects did not reach statistical significance. This suggests that while EVT alone may appear to favor these subgroups, further investigation with larger sample sizes may be needed to confirm the clinical relevance of these findings. Although studies suggested that patients with a BAO caused by cardio-embolism tend to have poorer responses to IVT compared to large-artery atherosclerosis BAOs, we did not objectivate this in our subgroup analyses [26, 27]. Identifying significant treatment effects among different subgroups may help to define the added value of IVT in specific patients. Therefore, the outcomes of this study should not discourage the use of IVT in BAO patients.

Several strengths and limitations of this study should be acknowledged. A strength is that this study is a pooled analysis of individual patient data from four international RCTs, enhancing the generalizability of the findings across a broad population and representing the highest quality data. However, centers participating in these four trials were dedicated stroke centers, potentially influencing the applicability in less experienced EVT stroke centers. Furthermore, there were no missing data for the primary outcome across any of the four trials. Missing data were most prevalent for recanalization rates in the BASICS trial. At baseline, information on occlusion location was the most frequently missing variable, because the BEST trial did not report this variable. A limitation is that patients, who improved after IVT before randomization did not enter one of the trials. Furthermore, patients included in this study were not randomized to either EVT with IVT or without IVT, introducing selection bias. Additionally, differences in patient selection criteria and varying enrollment periods among the trials may have introduced some experience and selection bias. Last, the low statistical power in subgroup analyses and variability in onset-to-randomization times (ranging from 0–6 to 24 h) could have influenced the results.

## Conclusions

Findings from this patient-level pooled analysis of four randomized controlled trials suggest that bridging IVT over EVT alone in BAO patients within 24 h after symptom onset was safe but not significant associated with improved outcomes.

## Supplementary Information

Below is the link to the electronic supplementary material.

Supplementary file1 (PDF 609 KB)

## References

[1] Berge E, Whiteley W, Audebert H, et al. European stroke organisation (ESO) guidelines on intravenous thrombolysis for acute ischaemic stroke. Eur Stroke J. 2021;6(1):I–LXII. 10.1177/2396987321989865. 33817340 10.1177/2396987321989865 PMC7995316

[2] Majoie CB, Cavalcante F, Gralla J, et al. Value of intravenous thrombolysis in endovascular treatment for large-vessel anterior circulation stroke: individual participant data meta-analysis of six randomised trials. Lancet. 2023;402:965–74. 10.1016/S0140-6736(23)01142-X. 37640037 10.1016/S0140-6736(23)01142-X

[3] Strbian D, Tsivgoulis G, Ospel J, et al. European Stroke Organisation (ESO) and European Society for Minimally Invasive Neurological Therapy (ESMINT) guideline on acute management of basilar artery occlusion. J Neurointerv Surg. 2024;16:e7. 10.1136/jnis-2024-022053. 39043395 10.1136/jnis-2024-022053 PMC11347260

[4] Knapen R, Frol S, van Kuijk SMJ, et al. Intravenous thrombolysis for ischemic stroke in the posterior circulation: a systematic review and meta-analysis. J Stroke Cerebrovasc Dis. 2024;33:107641. 10.1016/j.jstrokecerebrovasdis.2024.107641. 38395096 10.1016/j.jstrokecerebrovasdis.2024.107641

[5] Jovin TG, Li C, Wu L, et al. Trial of thrombectomy 6 to 24 hours after stroke due to basilar-artery occlusion. N Engl J Med. 2022;387:1373–84. 10.1056/NEJMoa2207576. 36239645 10.1056/NEJMoa2207576

[6] Langezaal LCM, van der Hoeven E, Mont’Alverne FJA, et al. Endovascular therapy for stroke due to basilar-artery occlusion. N Engl J Med. 2021;384:1910–20. 10.1056/NEJMoa2030297. 34010530 10.1056/NEJMoa2030297

[7] Tao C, Nogueira RG, Zhu Y, et al. Trial of endovascular treatment of acute basilar-artery occlusion. N Engl J Med. 2022;387:1361–72. 10.1056/NEJMoa2206317. 36239644 10.1056/NEJMoa2206317

[8] Liu X, Dai Q, Ye R, et al. Endovascular treatment versus standard medical treatment for vertebrobasilar artery occlusion (BEST): an open-label, randomised controlled trial. Lancet Neurol. 2020;19:115–22. 10.1016/S1474-4422(19)30395-3. 31831388 10.1016/S1474-4422(19)30395-3

[9] Nogueira RG, Jovin TG, Liu X, et al. Endovascular therapy for acute vertebrobasilar occlusion (VERITAS): a systematic review and individual patient data meta-analysis. Lancet. 2025. 10.1016/S0140-6736(24)01820-8. 39674187 10.1016/S0140-6736(24)01820-8

[10] Raty S, Virtanen P, Ritvonen J, et al. IV thrombolysis in basilar artery occlusion: outcomes and comparison with endovascular thrombectomy. Neurology. 2024;102:e209249. 10.1212/WNL.0000000000209249. 38531004 10.1212/WNL.0000000000209249

[11] Han B, Raynald, Wu Y, et al. Thrombectomy versus combined thrombolysis for acute basilar artery occlusion: a secondary analysis of the ATTENTION trial. J Neurointerv Surg. 2024. 10.1136/jnis-2024-021678. 10.1136/jnis-2024-021678 38937086

[12] Knapen R, Pirson FAV, Langezaal LCM, et al. Intravenous thrombolysis before endovascular treatment in posterior circulation occlusions: a MR clean registry study. Stroke. 2024;55:403–12. 10.1161/STROKEAHA.123.043777. 38174571 10.1161/STROKEAHA.123.043777 PMC10802980

[13] Cai L, Wang L, Campbell BCV, et al. Endovascular thrombectomy with versus without intravenous thrombolysis in patients with acute basilar artery occlusion: a systematic review and meta-analysis. J Neurol. 2024. 10.1007/s00415-024-12353-w. 38597945 10.1007/s00415-024-12353-w

[14] von Elm E, Altman DG, Egger M, et al. The strengthening the reporting of observational studies in epidemiology (STROBE) statement: guidelines for reporting observational studies. J Clin Epidemiol. 2008;61:344–9. 10.1016/j.jclinepi.2007.11.008. 18313558 10.1016/j.jclinepi.2007.11.008

[15] Mazya M, Egido JA, Ford GA, et al. Predicting the risk of symptomatic intracerebral hemorrhage in ischemic stroke treated with intravenous alteplase: safe Implementation of Treatments in Stroke (SITS) symptomatic intracerebral hemorrhage risk score. Stroke. 2012;43:1524–31. 10.1161/STROKEAHA.111.644815. 22442178 10.1161/STROKEAHA.111.644815

[16] Hacke W, Kaste M, Bluhmki E, et al. Thrombolysis with alteplase 3 to 4.5 hours after acute ischemic stroke. N Engl J Med. 2008;359:1317–29. 10.1056/NEJMoa0804656. 18815396 10.1056/NEJMoa0804656

[17] Strbian D, Sairanen T, Silvennoinen H, et al. Thrombolysis of basilar artery occlusion: impact of baseline ischemia and time. Ann Neurol. 2013;73:688–94. 10.1002/ana.23904. 23536323 10.1002/ana.23904

[18] Nappini S, Arba F, Pracucci G, et al. Bridging versus direct endovascular therapy in basilar artery occlusion. J Neurol Neurosurg Psychiatry. 2021;92:956–62. 10.1136/jnnp-2020-325328. 34035131 10.1136/jnnp-2020-325328

[19] Giorgianni A, Biraschi F, Piano M, et al. Endovascular treatment of acute basilar artery occlusion: registro endovascolare lombardo occlusione basilar artery (RELOBA) Study Group Experience. J Stroke Cerebrovasc Dis. 2018;27:2367–74. 10.1016/j.jstrokecerebrovasdis.2018.04.022. 29958848 10.1016/j.jstrokecerebrovasdis.2018.04.022

[20] Keselman B, Gdovinove Z, Jatuzis D, et al. Safety and outcomes of iv thrombolysis in posterior versus anterior circulation acute ischemic stroke. Results from the sits international stroke thrombolysis register (sits-ISTR) and meta-analysis. Euro Stroke J. 2019;4:110–11. 10.1177/2396987319845578.

[21] Lee SH, Han JH, Jung I, et al. Do thrombolysis outcomes differ between anterior circulation stroke and posterior circulation stroke? A systematic review and meta-analysis. Int J Stroke. 2020;15:849–57. 10.1177/1747493020909634. 32122288 10.1177/1747493020909634

[22] Sarikaya H, Arnold M, Engelter ST, et al. Outcomes of intravenous thrombolysis in posterior versus anterior circulation stroke. Stroke. 2011;42:2498–502. 10.1161/STROKEAHA.110.607614. 21778443 10.1161/STROKEAHA.110.607614

[23] Keselman B, Gdovinova Z, Jatuzis D, et al. Safety and outcomes of intravenous thrombolysis in posterior versus anterior circulation stroke: results from the safe implementation of treatments in stroke registry and meta-analysis. Stroke. 2020;51:876–82. 10.1161/STROKEAHA.119.027071. 31914885 10.1161/STROKEAHA.119.027071

[24] Vergouwen MDI, Algra A, Pfefferkorn T, et al. Time is brain(stem) in basilar artery occlusion. Cerebrovasc Dis. 2012;33:501–2. 10.1159/000339538. 22989501 10.1161/STROKEAHA.112.666867

[25] Puetz V, Sylaja P, Hill M, et al. CT angiography source images predict final infarct extent in patients with basilar artery occlusion. AJNR Am J Neuroradiol. 2009;30:1877–83. 19643923 10.3174/ajnr.A1723 PMC7051302

[26] Lee WJ, Jung KH, Ryu YJ, et al. Impact of stroke mechanism in acute basilar occlusion with reperfusion therapy. Ann Clin Transl Neurol. 2018;5:357–68. 10.1002/acn3.536. 29560380 10.1002/acn3.536 PMC5846447

[27] Cao J, Xing P, Zhu X, et al. Mild and moderate cardioembolic stroke patients may benefit more from direct mechanical thrombectomy than bridging therapy: a subgroup analysis of a randomized clinical trial (DIRECT-MT). Front Neurol. 2022. 10.3389/fneur.2022.1013819. 36504640 10.3389/fneur.2022.1013819 PMC9730510

