# Supplementary material for: Intravenous Thrombolysis Prior to Endovascular Treatment in Basilar Artery Occlusions: A Patient Pooled Analysis of Four Randomized Controlled Trials
Source: Cardiovasc Intervent Radiol. 2025 Oct 27;48(12):1869–77. doi: 10.1007/s00270-025-04251-z (PMC12665628; doi:10.1007/s00270-025-04251-z)
Supplement: Supplementary file 1 — Supplementary file1 (PDF 609 KB) [file 270_2025_4251_MOESM1_ESM.pdf]

Supplemental

Table of contents

Figure S1. Subgroup analyses on the primary outcome (mRS 0-3) p.2

Table S1. Outcomes of regression analyses on clinical, technical, and safety outcomes after IPTW p.4

Table S2. Sensitivity analysis in patients with onset to imaging <270 minutes of on clinical, technical, and safety outcomes after IPTW p.5

Table S3. Outcomes of regression analyses on clinical, technical, and safety outcomes after PSM p.6

Figure S1. Subgroup analyses on the primary outcome (mRS 0-3)

Subgroup	Total	aOR (95% CI)
Overall	556	0.84 (0.52 to 1.35)
Sex (p=0.29)		
Male	377	0.71 (0.42 to 1.22)
Female	179	1.44 (0.62 to 3.50)
Age (p=0.30)		
≤67	288	1.24 (0.67 to 2.27)
>67	268	0.56 (0.29 to 1.10)
Atrial fibrillation (p=0.69)		
Yes	121	0.66 (0.25 to 1.75)
No	435	0.92 (0.56 to 1.52)
Hypertension (p<0.01)		
Yes	391	0.67 (0.40 to 1.14)
No	165	1.41 (0.62 to 3.20)
NIHSS		
0-9 (ref)	68	0.35 (0.04 to 2.78)
10-20 (p=0.02)	187	1.69 (0.80 to 3.56)
>20 (p=0.18	301	0.56 (0.30 to 1.06)
Baseline mRS (p=0.18)		
0	469	0.94 (0.58 to 1.50)
≥1	86	0.51 (0.13 to 2.04)
TOAST		
LAA (ref)	273	1.00 (0.53 to 1.91)
CE (p=0.82)	123	0.63 (0.25 to 1.57)
SVD+other/undetermined (p=0.81)	152	0.73 (0.28 to 1.91)
Occlusion site		
Proximal 1/3 BA (ref)	156	0.33 (0.01 to 8.65)
Middle 1/3 BA (p=0.50)	151	0.79 (0.32 to 1.95)
Distal 1/3 BA (p=0.24)	137	1.90 (0.68 to 5.30)
Baseline PC-ASPECTS (p=0.86)		
0-7	107	0.86 (0.25 to 2.97)
8-10	437	0.84 (0.52 to 1.36)
Time to imaging		
<4.5h (ref)	303	1.15 (0.67 to 1.97)
4.5 - 6h (p=0.43)	76	0.39 (0.04 to 3.71)
>6h (p=0.27)	145	0.56 (0.13 to 2.48)

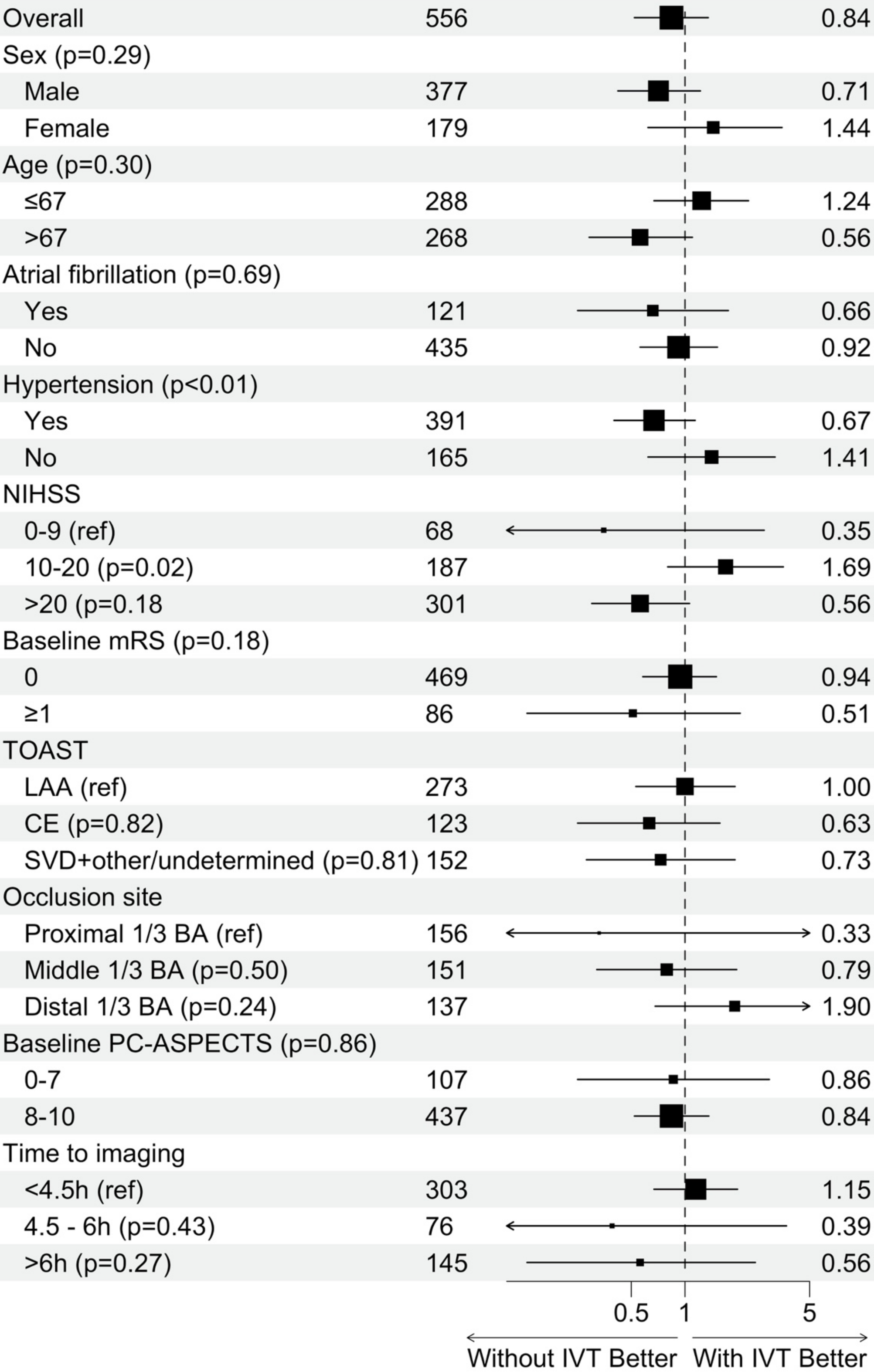

NIHSS, National Institutes of Health Stroke Scale; mRS, modified Rankin Scale; TOAST, Trial of Org 10172 in Acute Stroke Treatment; LAA, large vessel atherothromboembolic; CE, cardioembolic; SVD, small vessel

disease BA, basilar artery; pc-ASPECTS, posterior circulation Acute Stroke Prognosis Early Computed Tomography score.

Table S1. Outcomes of regression analyses on clinical, technical, and safety outcomes after IPTW

EVT alone as reference	EE	Unadjusted (95% CI)	Adjusted (95% CI)
**Primary outcome**			
mRS 0-3 at 90 days	OR	1.03 (0.73-1.45)	0.87 (0.60-1.26)
**Secondary outcomes**			
mRS at 90 days*	cOR	0.86 (0.61-1.21)	0.94 (0.67-1.33)
mRS 0-2 at 90 days	OR	1.41 (0.99-2.02)	1.23 (0.83-1.82)
NIHSS at 24-48 hours	ß	-2.48 (-5.04 to 0.09)	-0.69 (-3.03 to 1.65)
NIHSS >10 improvement	OR	**0.68 (0.47-0.97)**	0.75 (0.51-1.09)
Post mTICI ≥2B	OR	0.80 (0.51-1.25)	0.80 (0.51-1.27)
Post mTICI 3	OR	1.31 (0.86-1.97)	1.29 (0.85-1.97)
**Safety outcomes**			
Mortality at 90 days	OR	0.90 (0.62-1.29)	1.01 (0.69-1.49)
Symptomatic ICH	OR	1.71 (0.77-3.79)	1.71 (0.77-3.79)

*: shift towards a higher (worse) functional outcome on the full scale.

EE, effect estimate; OR, odds ratio, cOR, common odds ratio; mRS, modified Rankin Scale; eTICI, extended Thrombolysis In Cerebral Infarction; ICH, intracranial hemorrhage; NIHSS, National Institutes of Health Stroke Scale.

Table S2. Sensitivity analysis in patients with onset to imaging <270 minutes of on clinical, technical, and safety outcomes after IPTW

EVT alone as reference	EE	Unadjusted (95% CI)	Adjusted (95% CI)
**Primary outcome**			
mRS 0-3 at 90 days	OR	1.18 (0.74-1.87)	1.03 (0.63-1.69)
**Secondary outcomes**			
mRS at 90 days*	cOR	0.77 (0.54-1.12)	0.84 (0.57-1.21)
mRS 0-2 at 90 days	OR	1.58 (0.97-2.57)	1.41 (0.83-2.40)
NIHSS at 24-48 hours	ß	-2.77 (-6.18 to 0.65)	-1.28 (-4.45 to 1.90)
NIHSS >10 improvement	OR	0.76 (0.47-1.22)	0.83 (0.51-1.37)
Post mTICI ≥2B	OR	0.96 (0.51-1.81)	0.98 (0.51-1.86)
Post mTICI 3	OR	1.51 (0.83-2.73)	1.54 (0.84-2.82)
**Safety outcomes**			
Mortality at 90 days	OR	0.79 (0.49-1.29)	0.87 (0.52-1.46)
Symptomatic ICH	OR	1.61 (0.54-4.76)	1.55 (0.54-4.49)

*: shift towards a higher (worse) functional outcome on the full scale.

IPTW, inverse probability of treatment weighting; EE, effect estimate; OR, odds ratio, cOR, common odds ratio; mRS, modified Rankin Scale; eTICI, extended Thrombolysis In Cerebral Infarction; ICH, intracranial hemorrhage; NIHSS, National Institutes of Health Stroke Scale.

Table S3. Outcomes of regression analyses on clinical, technical, and safety outcomes after PSM

EVT alone as reference	EE	Unadjusted (95% CI)	Adjusted (95% CI)
**Primary outcome**			
mRS 0-3 at 90 days	OR	1.21 (0.72-2.05)	1.05 (0.59-1.88)
**Secondary outcomes**			
mRS at 90 days*	cOR	0.78 (0.49-1.24)	0.83 (0.51-1.35)
mRS 0-2 at 90 days	OR	1.47 (0.84-2.59)	1.31 (0.70-2.43)
NIHSS at 24-48 hours	ß	-3.65 (-7.62 – 0.31)	-1.86 (-5.44 - 1.72)
NIHSS >10 improvement	OR	0.83 (0.46-1.50)	0.92 (0.50-1.69)
Post mTICI ≥2B	OR	0.83 (0.41-1.69)	0.82 (0.40-1.70)
Post mTICI 3	OR	0.99 (0.51-1.94)	0.98 (0.49-1.95)
**Safety outcomes**			
Mortality at 90 days	OR	0.76 (0.44-1.32)	0.84 (0.46-1.53)
Symptomatic ICH	OR	1.83 (0.46-7.25)	1.92 (0.48-7.70)

*: shift towards a higher (worse) functional outcome on the full scale.

PSM, propensity score matching; EE, effect estimate; OR, odds ratio, cOR, common odds ratio; mRS, modified Rankin Scale; eTICI, extended Thrombolysis In Cerebral Infarction; ICH, intracranial hemorrhage; NIHSS, National Institutes of Health Stroke Scale.
